# Distributed Acoustic Sensing Based on Coherent Microwave Photonics Interferometry

**DOI:** 10.3390/s21206784

**Published:** 2021-10-13

**Authors:** Liwei Hua, Xuran Zhu, Baokai Cheng, Yang Song, Qi Zhang, Yongji Wu, Lawrence C. Murdoch, Erin R. Dauson, Carly M. Donahue, Hai Xiao

**Affiliations:** 1 Holcombe Department of Electrical and Computer Engineering, Clemson University, Clemson, SC 29634, USA; lhua@g.clemson.edu (L.H.); xuranz@g.clemson.edu (X.Z.); baokaic@g.clemson.edu (B.C.); song5@g.clemson.edu (Y.S.); qzhang9@g.clemson.edu (Q.Z.); yongjiw@g.clemson.edu (Y.W.); 2 Department of Environmental Engineering and Earth Sciences, Clemson University, Clemson, SC 29634, USA; lmurdoc@clemson.edu; 3 Geophysics Group, Los Alamos National Laboratory, Los Alamos, NM 87545, USA; erindauson@lanl.gov (E.R.D.); cmd@lanl.gov (C.M.D.)

**Keywords:** fiber optics sensors, microwave photonics, interferometry, distributed acoustic sensing (DAS), optical frequency domain reflectometry (OFDR)

## Abstract

A microwave photonics method has been developed for measuring distributed acoustic signals. This method uses microwave-modulated low coherence light as a probe to interrogate distributed in-fiber interferometers, which are used to measure acoustic-induced strain. By sweeping the microwave frequency at a constant rate, the acoustic signals are encoded into the complex microwave spectrum. The microwave spectrum is transformed into the joint time–frequency domain and further processed to obtain the distributed acoustic signals. The method is first evaluated using an intrinsic Fabry Perot interferometer (IFPI). Acoustic signals of frequency up to 15.6 kHz were detected. The method was further demonstrated using an array of in-fiber weak reflectors and an external Michelson interferometer. Two piezoceramic cylinders (PCCs) driven at frequencies of 1700 Hz and 3430 Hz were used as acoustic sources. The experiment results show that the sensing system can locate multiple acoustic sources. The system resolves 20 nε when the spatial resolution is 5 cm. The recovered acoustic signals match the excitation signals in frequency, amplitude, and phase, indicating an excellent potential for distributed acoustic sensing (DAS).

## 1. Introduction

Distributed acoustic sensing (DAS) employs optical fiber to acquire many acoustic signals using one interrogation unit. Optical fibers are small, have low loss, and are resistant to corrosion and electromagnetic interference, so they can be easily embedded into structures and function in harsh environments. Thus, DAS has become a powerful tool to understand geodynamics [1,2], and it also provides safety and integrity monitoring solutions in the fields of transportation [3], oil and gas [4], civil infrastructures [5], and related. These applications have diverse requirements in spatial resolution, measurement range, sensing bandwidth, and sensitivity. This has led to two main approaches, one involving DAS measurements in the time domain and another in the frequency domain [6].

The time-domain approaches, including phase-sensitive optical time-domain reflectometry (Φ-OTDR) and related [7,8,9], work by sending light pulses into fiber and collecting the backscattered light in the time domain. They have the advantages of high sensitivity, real-time detection, and long measurement range [9,10]. Methods such as data averaging, optical pulse coding [11], coherence detection [12], chirped pulse amplification [13], etc., have been developed to increase the signal-to-noise ratio (SNR) and spatial resolution. However, as the light pulse energy is proportional to the pulse width and positively related to the SNR, the time-domain approaches have inherent tradeoffs between spatial resolution, sensing range, measurement accuracy, and measurement time [14]. Typically, the acoustic detection band of Φ-OTDR ranges from tens of kiloherz for a few kilometers to hundreds of herz for more than 100 km with meter-level spatial resolution [15].

Frequency-domain approaches based on optical frequency-domain reflectometry (OFDR) offer higher spatial resolution without impairing the SNR [16]. In a typical OFDR system, a strong coherent, continuous lightwave is used as the probe. The center wavelength of the light is swept over a certain wavelength band. The spatial resolution is inversely proportional to the frequency sweep range, which can reach the submillimeter scale [17]. Recent developments of OFDR for DAS have focused on increasing the sweep repetition rate [18], decreasing the computational complexity [16,19], and suppressing crosstalk among sensors [20]. A bandwidth of more than a kilohertz was achieved using several different OFDR methods [18,20]. However, the measurement range of conventional OFDR systems is significantly shorter than the time-domain methods due to coherence fading and polarization fading. This has led to intricate polarization diversity detection methods, but even with these techniques, the typical measurement range for OFDR DAS is less than several kilometers [20,21].

Microwave photonics technologies have been investigated for distributed sensing in recent years [22,23,24,25]. The sensing systems use microwave-modulated light as the probe, and the backscattering from the sensing fiber is recorded in the microwave frequency domain through down conversion. The highly stable microwave phase determines locations along the signal path and thus enables a measurement range of more than 10 km [26]. Frequency modulation in the gigaherz bandwidth enables spatial resolution in the centimeter range [27]. Distributed systems based on this approach have been developed for interrogating Rayleigh scattering [28], fiber Bragg grating (FBG) arrays [29,30,31], and interferometers [24,27]. Recently, coherence-length-gated microwave photonics interferometry (CMPI) was developed to improve strain sensitivity using cascaded interferometers [24,32]. The coherence length of the light probe is well controlled to enable localized high-contrast optical interference while avoiding crosstalk among interferometers. The in-fiber ultra-fast laser-inscribed weak reflector arrays are used to form the interferometers, which has been demonstrated to significantly reduce interference fading and enhance the SNR in a distributed sensing system [33,34]. However, most of the above-mentioned systems require scanning through the entire microwave band to acquire one frame of distributed strain information, and this limits the measurement bandwidth to less than 100 Hz. Recent investigations show that microwave multitone modulation [35] or sparse frequency measurements [30] can significantly improve the measurement rate, resulting in tens of kilohertz of sensing bandwidth. However, either the sensing range or the number of sensing units is limited by those approaches.

The objective of this paper is to describe a new method for DAS measurements made with CMPI. This method records the strains caused by acoustic signals during the microwave frequency scanning and separates the strain signals in space through a time–frequency joint approach. As a result, an acoustic detection bandwidth of tens of kilohertz for distributed sensing with centimeter-scale spatial resolution can be obtained. The bandwidth has been increased more than a thousand times compared to our former reported microwave photonics distributed sensing systems [24,27,36].

## 2. Mathematical Model

**Figure 1:**
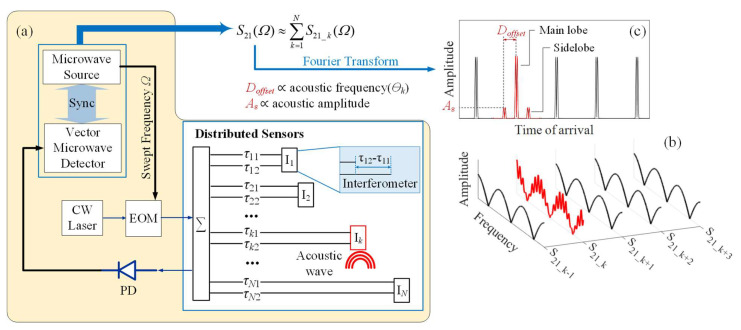
(**a**) Schematic illustration of the CMPI system for acoustic distributed acoustic sensing. PD: photodetector; EOM: electro-optic modulator; CW laser: continuous-wave laser. (**b**) The amplitude of the decomposed frequency-domain signal from each interferometer. (**c**) The amplitude of the time-domain signal.

A mathematical model of the CMPI system represents its main components, including a microwave source, a vector microwave detector, a light source, an electro-optical modulator, a photodetector (PD), and the distributed sensors formed by the interferometers (Figure 1a). A continuous-wave (CW) laser with a center frequency of *ω* is used as the light source. The light intensity is modulated by a microwave signal. The light launches into an optical fiber sensing network and is reflected by reflectors with respective time delays *τ*. Any two reflections that have a lag (*τ_k2_-τ_k1_*) much smaller than the coherence time of the laser produce optical interference, so the responsive light paths form an interferometer I*_k_*. Light reflections are injected into a PD, which converts the light power into electric voltage and sends it back to the vector microwave detector. The frequency of the microwave signal is swept over a designed range. At each modulation frequency *Ω*, synchronized detection is conducted, and the amplitude variation and phase shift of the modulation envelope are measured.

We designed the optical path difference (OPD) for each interferometer to be much smaller than the coherence length of the light source. The separation distance between adjacent interferometers can also be designed to be much larger than the laser coherence length, so the optical interference only occurs within each interferometer, and it is negligible among different interferometers. The sensing network system is equivalent to a linear combination of independent interferometers *I_k_* (*k* = 1, 2,…, *N*). Thus, the complex frequency response of the system *S*_21_ can be approximately expressed as:

(1) $$S_{21}(\Omega )\approx rect\left(\frac{\Omega -\Omega_{c}}{B_{\Omega }}\right)\cdot \sum_{k=1}^{N}S_{21\_k}(\Omega ),$$

where *B_Ω_* and *Ω_c_* are the bandwidth and center frequency of the microwave signal, respectively, and $S_{21\_k}(\Omega )$ is the frequency response from the *k*th interferometer, which is expressed as [24,32]:

(2) $$S_{21\_k}\left(\Omega \right)=m\left[\left(A_{k1}{}^{2}+A_{k1}A_{k2}\cos\Delta \phi_{k}\right)e^{-j\tau_{k1}\Omega }+\left(A_{k2}{}^{2}+A_{k1}A_{k2}\cos\Delta \phi_{k}\right)e^{-j\tau_{k2}\Omega }\right],$$

where *m* is a constant decided by the modulation depth of the EOM; *A_k_*_1_ and *A_k2_* are the amplitudes of the two reflected waves; $\Delta \phi_{k}$ is the optical phase difference between the two waves.

If the OPD of the two reflected waves in the *k*th interferometer (*L_k_*) changes sinusoidally as a function of time, then the optical phase difference varies accordingly and is expressed as:

(3) $$\Delta \phi_{k}=\left[L_{k}+\delta_{k}\cos(\Theta_{k}t+\Phi_{0})\right]\omega /c,$$

where $\Theta_{k}$ and $\delta_{k}$ are the frequency and amplitude of the dynamic OPD changes, respectively, and t is the time variable.

If the microwave modulation frequency is linearly scanned with a step of ΔΩ and a constant sampling time Δt, we can express *t* as:

(4) $$t=\left(\Omega -\Omega_{start}\right)/\Delta \Omega \cdot \Delta t,$$

where $\Omega_{start}$ is the first frequency within the microwave scanning band.

By applying the Fourier-Bessel series, $\cos\Delta \phi_{k}$ can be expressed as:

(5) $$\cos\Delta \phi_{k}=J_{0}\left(\frac{\delta_{k}\omega }{c}\right)\cos\left(\frac{L_{k}\omega }{c}\right)+2\sum_{m=1}^{\infty }J_{m}\left(\frac{\delta_{k}\omega }{c}\right)\cdot \cos\left(\frac{L_{k}\omega }{c}+\frac{m\pi }{2}\right)\cos\left[m\left(\frac{\Theta_{k}\Omega }{\Delta \Omega }\Delta t+\Phi_{cons}\right)\right],$$

where:

(6) $$\Phi_{cons}=\Phi_{0}-\Theta_{k}\Omega_{start}/\Delta \Omega \cdot \Delta t.$$

When the amplitude of the dynamic OPD change is much smaller than 2π times the wavelength of the light carrier, i.e., $\delta_{k}\ll c/\omega$, Equation (5) can be approximated by keeping the low order (*J*_0_ and *J*_1_) Bessel terms. The linear approximation is expressed as:

(7) $$\cos\Delta \phi_{k}\approx \cos\frac{L_{k}\omega }{c}-\frac{\delta_{k}\omega }{c}\sin\frac{L_{k}\omega }{c}\cos\left(\frac{\Theta_{k}\Omega }{\Delta \Omega }\Delta t+\Phi_{cons}\right)$$

Substituting Equation (7) into Equation (2), the result indicates that the dynamic OPD change with the frequency $\Theta_{k}$ is modulated on the amplitude of the microwave interferogram $S_{21\_k}$, as illustrated in Figure 1b. The complex inverse Fourier transform of the $S_{21}(\Omega )$ can be expressed as:

(8) $$F(t_{z})=\left[mB_{\Omega }\sin c\left(B_{\Omega }t_{z}\right)e^{-j\Omega_{c}t_{z}}\right]*\sum_{k=1}^{N}F_{k}(t_{z}),$$

which includes “Static” and “Dynamic” components as:

(9) $$\begin{array}{l} F_{k}\;(t_{z})={\left\{\left[A_{k1}{}^{2}+A_{k1}A_{k2}\cos\frac{L_{k}\omega }{c}\right]\delta (t_{z}-\tau_{k1})+\left[A_{k2}{}^{2}+A_{k1}A_{k2}\cos\frac{L_{k}\omega }{c}\right]\delta \left(t_{z}-\tau_{k2}\right)\right\}}_{\mathrm{Static}}\\ -{\left\{\begin{array}{r} \frac{\delta_{k}\omega }{c}A_{k1}A_{k2}\sin\frac{L_{k}\omega }{c}\cdot \left[\delta \left(t_{z}-\tau_{k1}+\Theta_{k}\frac{\Delta t}{\Delta \Omega }\right)e^{j\Phi_{cons}}+\delta \left(t_{z}-\tau_{k1}-\Theta_{k}\frac{\Delta t}{\Delta \Omega }\right)e^{-j\Phi_{cons}}\right]\\ +\frac{\delta_{k}\omega }{c}A_{k1}A_{k2}\sin\frac{L_{k}\omega }{c}\cdot \left[\delta \left(t_{z}-\tau_{k2}+\Theta_{k}\frac{\Delta t}{\Delta \Omega }\right)e^{j\Phi_{cons}}+\delta \left(t_{z}-\tau_{k2}-\Theta_{k}\frac{\Delta t}{\Delta \Omega }\right)e^{-j\Phi_{cons}}\right] \end{array}\right\}}_{\mathrm{Dynamic}} \end{array}$$

The “Static” component in Equation (9) includes two time-pulses (main lobes) that are generated by the two reflected waves from interferometer I*_k_*, and they are centered at and, respectively. The “Dynamic” component is formed by time pulses generated by the dynamic OPD changes. The dynamic pulse pairs behave as sidelobes, which occur at both sides of the respective main lobes (Figure 1c).

The offset between a sidelobe and the corresponding main lobe (*D_offset_*) is directly proportional to the acoustic frequency $\Theta_{k}$ with a scaling coefficient Δt/ΔΩ. The amplitude of the dynamic OPD change $\delta_{k}$ is directly proportional to the amplitude of the sidelobes.

We calculate $\delta_{k}$ by using the sidelobe amplitude and the interference phase $L_{k}\omega /c$. The interference phase is calculated using the chirp effect of the EOM to perform quadrature-phase demodulation [32]. This is done by tuning the EOM bias to add opposite phase shifts of π/4 to the optical interference phase, so the peak values of the two main lobes are in quadrature as $A_{k1}{}^{2}+A_{k1}A_{k2}\cos\left(L_{k}\omega /c+\pi /4\right)$ and $A_{k2}{}^{2}+A_{k1}A_{k2}\cos\left(L_{k}\omega /c-\pi /4\right)$. The standard quadrature-phase demodulation method can be used to calibrate the conic coefficients ($A_{k1}{}^{2}$,$A_{k2}{}^{2}$$A_{k1}A_{k2}$, and the quadrature error) and calculate the interference phase [37].

We extract $S_{21\_k}\left(\Omega \right)$ from $S_{21}\left(\Omega \right)$ through an inverse Fourier transform of the gated time-domain signal as:

(10) $$S_{21\_k\_g}\left(\Omega \right)=ℱ\left[F(t_{z})\cdot g_{k}(t_{z})\right].$$

where *g_k_*(*t_z_*) is a time-domain gate function that selects the time pulses generated by the *k*th interferometer.

The temporal signal can be reconstructed by applying a high-pass filter to $\left|S_{21\_k\_g}\left(\Omega \right)\right|$. The time is linearly converted from *Ω* using Equation (4), and the amplitude is also corrected by using the calculated interference phase.

## 3. Experiment

Several ways to build a sensing network using the CMPI system have been demonstrated [24,37], and we used two of these methods to validate the proposed concept. The first set of experiments used a single pair of in-fiber reflectors to illustrate the signal processing. The next set of experiments used an array of in-fiber reflectors and an external Michelson interferometer (MI) to verify distributed acoustic sensing. The reflectors used in the experiments were fabricated using femtosecond laser micromachining, which created reflectivity from −35 to −45 dB [38].

### 3.1. System Configuration

A single longitude mode of the F-P laser (HP81554, Hewlett-Packard, USA) is filtered out by a bandpass filter (BPF1) and used as the light carrier. The selected mode has a center wavelength of 1543 nm and a coherence length of 6 cm. The light was intensity-modulated by a microwave signal via an EOM (Lucent, X2623Y, Murray Hill, NJ, USA). The modulation signal was generated by a vector network analyzer (VNA, Agilent E8364B, Santa Clara, CA, USA). The bias voltage of the EOM was provided by an external DC power supply. An inline polarization controller was used to optimize the modulation depth of the EOM. The microwave-modulated light output from the EOM was first amplified by an erbium-doped fiber amplifier (EDFA)1 and then launched into the distributed sensors. Reflected signals from the distributed fiber sensors were amplified by the EDFA2 and filtered by the BPF2. A high-speed PD detected the filtered signal and passed over the converted electrical signal to VNA through port 2.

### 3.2. Frequency and Amplitude Reading

**Figure 2:**
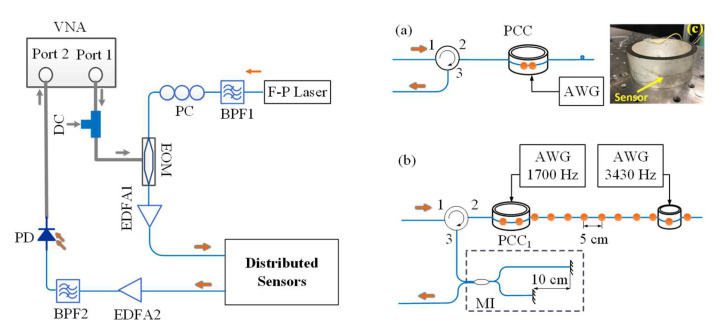
Schematic of the experimental setup for DAS by using CMPI. The optical fiber sensing network was formed by (**a**) a single reflector pair and (**b**) a weak reflector array and a Michelson interferometer. (**c**) Optical fiber with weak reflectors taped around the PCC. PCC: piezo ceramic cylinder; AWG: arbitrary waveform generator, EDFA: erbium-doped fiber amplifier.

In this experiment, the sensor was formed by two weak reflectors with a separation distance of 1 cm. The fiber sensor was taped on a piezoceramic cylinder (PCC) (dim. Ø85 × 32 mm), as shown in Figure 2a,c. The PCC was driven by an arbitrary waveform generator (AWG, Agilent 33120A, Santa Clara, CA, USA) to generate acoustic signals. The VNA was set to have 16,001 sampling points in the microwave band from 1 GHz to 1.1 GHz, enabling a 16 km interrogation range and 1 m spatial resolution. Therefore, the reflection from the two reflectors was not separable and was shown as a single pulse at the sensor location (23.6 m) in the time domain. We set the IFBW to 35 kHz to obtain a 30 μs (Δt=30 μs) sampling time at each modulation frequency.

**Figure 3:**
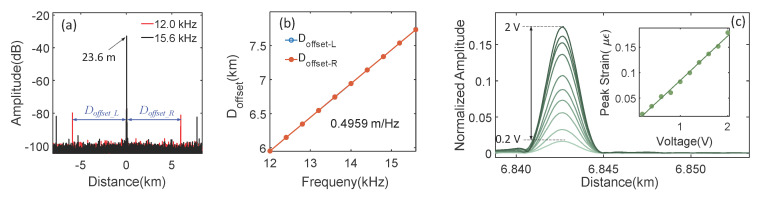
(**a**) Time-domain signals when 12 kHz and 15.6 kHz acoustic signal are generated by the PCC. (**b**) The offset between the main lobe and sidelobes as a function of acoustic frequency. (**c**) Right sideband of the first-order harmonic at the different driving voltages for acoustic frequency of 13.75 kHz. Inset: Peak strain as a function of the driving voltage.

A sinusoid signal with an amplitude of 1 V was used to drive the PCC. The signal frequency was swept from 12 kHz to 15.6 kHz with a step of 400 Hz. *S*_21_ was recorded at each step, and time-domain signals were obtained by applying the Fourier transform. Figure 3a shows the amplitude of the time-domain signals when 12 kHz and 15.6 kHz acoustic signals were generated by the PCC. The main lobe is located at 23.6 m. The acoustic-wave-generated first-order sidelobes are at (−5.9272 km, 5.9725 km) and (−7.7124 km, 7.7597 km) when 12 kHz and 15.6 kHz acoustic signals were excited, respectively. The offset between the main lobe and both sidelobes (*D*_offset-L,_ *D*_offset-R_) increased linearly as the acoustic frequency increased, as shown in Figure 3b. The slope of the linear fitting line is 0.4959 m/Hz (a refractive index of 1.452 was used in the calculation), which is the same as the estimated value from Equation (4). The norm of the residual of the linear fitting is 0.1 mm/Hz.

The acoustic frequency was then fixed at 13.75 kHz, and the amplitude of the acoustic signal was tuned by tuning the driving voltage from 0.2 V to 2 V with 0.2 V per step. The amplitude of the right-sidelobe increased as the driving voltage increased, as shown in Figure 3b. The amplitudes of the strains (ε) induced by the acoustic signal were calculated through the peak values of the main lobe and sidelobes. The results show that the peak strain increased linearly as a function of the applied voltage (inset of Figure 3b).

### 3.3. Temporal Signal Reconstruction

**Figure 4:**
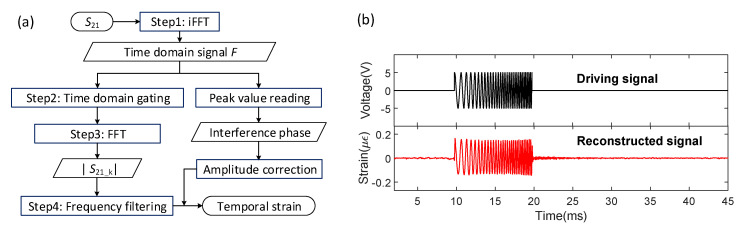
(**a**) Signal flow chart for temporal signal reconstruction. (**b**) Driving and reconstructed chirp signals.

The phase information of the acoustic wave is retained in the time-domain signal, according to Equation (9). Therefore, the proposed method can be used to reconstruct the temporal signal at each sensor location. The reconstruction procedure requires four steps as follows (Figure 4a):

Step 1: Apply a Fourier transform to *S*_21_ to obtain the time-domain signal.

Step 2: Apply the time-domain gate that is used to filter out the main lobe and sidelobes of the *k*th sensor.

Step 3: Apply the inverse Fourier transform to the filtered time-domain signal to construct *S*_21___k_ for each sensor.

Step 4: Apply the high-pass filter to |*S*_21___k_| to get the reconstructed temporal signal with normalized amplitude. The actual amplitude of the temporal signal can be calculated by using the peak values of the respective time-domain pulses.

We demonstrated the temporal signal reconstruction by using a frequency-chirped pulse. The pulse was triggered by the VNA and applied to the PCC. The pulse width was 10 ms, with a linear variation of the instantaneous frequency from 1 kHz to 5 kHz. The separation distance between reflectors was increased from 1 cm to 15 cm to increase the strain sensitivity. The F-P laser was substituted by a distributed feedback (DFB) laser (2 MHz linewidth), so the coherence length of the light source was much longer than the OPD of the sensor cavity. The VNA was set to have 1601 sampling points in the microwave band from 1 GHz to 1.3 GHz. The test was repeated ten times, and the VNA started scanning 9.781 ms before the pulse was generated. The temporal signal reconstruction was performed by following the steps shown in Figure 4a. A Tukey window with a cosine fraction of 0.7 was used in Step 2. A second-order Butterworth high-pass filter with a stop frequency of 500 Hz was used in Step 4.

The driving signal and the averaged reconstructed temporal signal are in phase, and their frequency components are nearly identical (Figure 4b). Amplitudes are also nearly identical using a scaling of 0.03 με/V. The high-frequency ripples after 20 ms are only showing in the reconstructed temporal signal, which could be acoustic resonance excited by the acoustic pulse. These results demonstrate that the temporal acoustic signal waveform can be detected by using this method with high fidelity.

### 3.4. Distributed Measurement by Using an Array of Reflectors

Multiple weak reflectors can be cascaded in fiber to create a distributed CMPI sensor. However, dark zones (low-sensitivity regions) need to be inserted between adjacent reflector pairs to avoid the crosstalk among interferometers. One way to avoid dark zones is to fabricate reflectors with similar intervals and use an external Michelson interferometer (MI) in the fiber sensing network [36]. The light is reflected by the reflectors and then coupled into the MI. The coherence length of the light source is much smaller than the intervals of cascaded reflectors. Thus, only the reflector pairs with separation distance close to the arm length difference of the MI (*L*) are selected out to form the interferometers. The strain changes between selected reflector pairs can be measured using this system.

We used an array of 12 weak reflectors 5 cm apart on a piece of SMF and an optical-fiber-based MI (Figure 2b) to demonstrate the idea. The MI was made with a 1 × 2 SMF 50:50 coupler and two Faraday rotation mirrors (FRM). The MI was embedded in a block of epoxy to reduce the OPD change induced by environmental influences (temperature, vibration, etc.). The light was reflected by the reflectors and then reflected by the MI. The coherence length of the light source is shorter than two times the optical interval of the reflectors, i.e., 6 cm < 2·*n·*5 cm, so only the reflector pairs with separation distance close to the arm length difference of the MI are selected to form the interferometers. For example, the light reflected by the first reflector and longer arm of MI would produce strong interference with the light reflected by the third reflector and shorter arm of MI. The strain changes between first and third reflectors (second and fourth, third and fifth, and so on) can be measured using this system. Therefore, 10 sensing units along the SMF were formed for spatially continuous monitoring.

Two piezoceramic cylinders (PCCs) were used to produce acoustic signals at two different locations. A 5 cm long fiber section centered at 37.05 m (between the first and second reflector) was taped in the tangential direction along the outer surface of PCC_1_ (dim Ø85 × 32 mm), and another 5 cm long fiber section centered at 37.47 m (around the 11th reflector) was taped along the outer surface of PCC_2_ (dim Ø47 × 40 mm) as shown in Figure 2. The fiber between the two PCCs was freely placed on an optical table and did not substantially couple acoustic waves. Two AWGs were used to provide driving signals to the PCCs. The driving signals were synchronized and triggered by the VNA. Two driving signals with frequencies of 1700 Hz and 3430 Hz were applied to the PCC_1_ and PCC_2,_ respectively.

**Figure 5:**
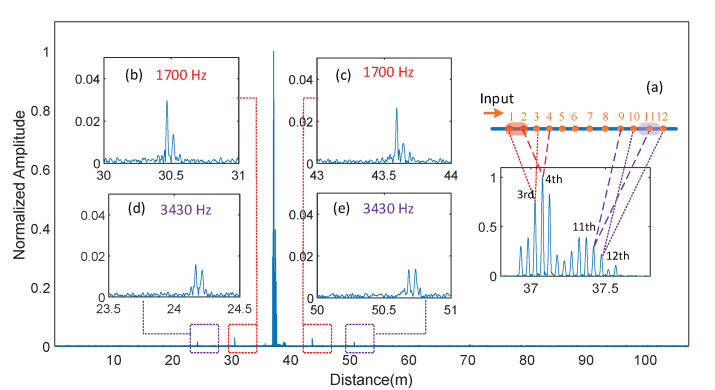
Time-domain signal when the PCCs located at 37.05 m and 37.47 m were driven at 1700 Hz and 3430 Hz. Insets: Expanded time signal. (**a**) The main lobes formed by the reflector array; (**b**,**c**) sidelobes generated by the PCC1 centered at 37.43 m and driven at 1700 Hz; (**d**,**e**) sidelobes caused by the vibration of PCC2 located at 37.08 m and driven at 3430 Hz.

**Figure 6:**
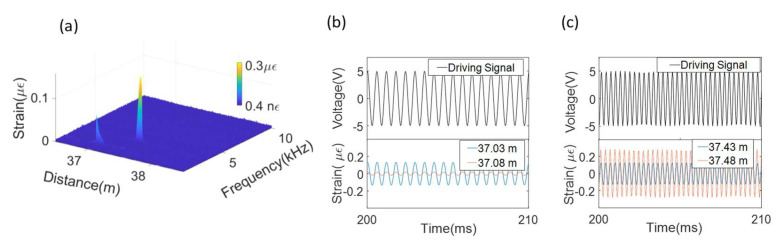
(**a**) Strain as functions of frequency and distance. (**b**) Driving signal (upper) to PCC_1_ and the reconstructed temporal signals centered at 37.03 m and 37.08 m (lower). (**c**) Driving signal (upper) to PCC_2_ and the reconstructed temporal signals centered at 37.43 m and 37.48 m (lower).

The VNA measured the amplitude and phase of the electrical signal at the modulation frequency, which was set to 16,001 sampling points in the microwave band from 0.1 GHz to 15.1 GHz. As the sensing range is inversely proportional to the sampling interval, the maximum sensing range was reduced to 80 m. The IFBW was set to 30 kHz, so the average sampling time for each sampling point was 0.036 ms (Δt=0.036ms). The bandwidth for each sensing section is solely dependent on the IFBW, which was 13.8 kHz for each sensing unit. In the time domain, the pulse reflected by the *k*th reflector (*k* < 11) and the long arm of the MI were overlapped with the pulse that was reflected by the (*k* + 2)th reflector and the short arm of the MI. Therefore, 14 pulses were formed within the range of 36.93 m to 37.57 m, as shown in Figure 5a. The peak value of the 3rd, 4th, 11th, and 12th pulses was used to read the interference phase from the interferometers formed by the (1,3), (2,4), (9,11), and (10,12) reflectors, respectively. The four small pulse clusters highlighted by the dashed line boxes in Figure 5 were the sidelobes generated by the vibration from the PCCs. The two pairs of sidelobes located around 30.50 m (Figure 5b) and 43.61 m (Figure 5c) were symmetrically distributed to the third and fourth pulses, respectively. The two pairs of sidelobes located around 24.18 m (Figure 5d) and 50.72 m (Figure 5e) were symmetrically distributed to the 11th and the 12th pulses, respectively. The vibration locations and frequencies were clearly distinguished, as shown in Figure 6a, where the processed strain was plotted as a function of frequency and distance.

The temporal signal reconstruction was performed by following the steps shown in Figure 4a. Time-domain gates that truncated the main pulse and the respective sidelobe at 1700 Hz and 3430 Hz were applied to the four affected sensing units separately in Step 2. The reconstructed temporal signals from the sensing units centered at 37.03 m and 37.08 m were both in phase with the driving signal to PCC_1,_ as shown in Figure 6b. The reconstructed temporal signals centered at 37.43 m and 37.48 m were both in phase with the driving signal to PCC_2,_ as shown in Figure 6c. The strain amplitude was proportional to the contact length between the sensor unit and their respective PCC. The strain amplitude read from the sensor unit centered at 37.03 m was larger than that at 37.08 m, as the PCC_1_ was attached to the fiber section from 36.98 m to 37.03 m. Likewise, the strain amplitude read from the sensor unit centered at 37.48 m was larger than that at 37.43 m, as more than half of the taped fiber section to PCC_2_ was after 37.48 m.

The results show that the phase and amplitude of the acoustic signal are well resolved in the reconstructed temporal signals. The amplitude of 19.5 nε at 37.08 m was clearly identified, indicating a lower detection limit, less than that for the experiment setting. The strains from the sensor units that are 5 cm apart are distinguishable, demonstrating a spatial resolution of 5 cm for DAS.

Note that there are three terms to describe the range resolution: spatial resolution (*S_R_*), range sampling resolution ($S_{r}$), and gauge length (*L_G_*). In this paper, *S_R_* refers to the separation distance between the adjacent sensing units; *S_r_* refers to the sampling spacing after Fourier transform (Sr=c2BΩn); *L_G_* refers to the arm length difference of the MI. Therefore, in this experiment, the respective values for *S_R_*, *Sr*, and *L_G_* are 5 mm, 6.7 mm, and 10 cm, respectively.

## 4. Discussion

### 4.1. Maximum Measurable Frequency vs. Reception Bandwidth

Since the acoustic signal is sampled during microwave frequency scanning, the sampling rate is determined by the frequency scanning sampling time (Δt). Therefore, the maximum measurable frequency is expressed as $\Theta_{max}=1/2\Delta t$. In experiments, we showed that $\Theta_{max}$ equals 16.6 kHz when the IFBW is set as 35 kHz (Section 3.2 and Section 3.3) and $\Theta_{max}$ equals 13.8 kHz when the IFBW is set as 30 kHz (Section 3.4). An acoustic signal centered at any frequency between DC and $\Theta_{max}$ could be detected.

**Figure 7:**
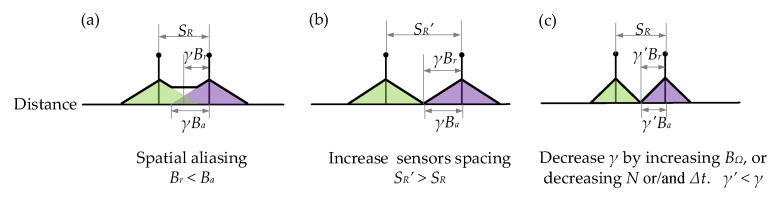
Relationship between the spatial resolution (*S_R_*) and reception bandwidth (*B_r_*)**.** (**a**) Spatial aliasing when *S_R_ < 2γ B_a_*. (**b**) Increase *S_R_* or (**c**) decrease *γ* to avoid spatial aliasing.

However, the sidelobes generated by different sensors may overlap when the acoustic signal bandwidth (*B_a_*) approaches the upper limit, as shown in Figure 7a. These overlaps (spatial aliasing) cause crosstalk among sensors, and more sophisticated signal processing methods are required to identify the frequency components and recover the temporal signal. We call the bandwidth that avoids spatial aliasing the reception bandwidth (*B_r_*), which can be expressed as:

(11) $$B_{r}=\frac{S_{R}}{2\gamma },\;\mathrm{where}\;\gamma =\frac{cN\Delta t}{2B_{\Omega }n}.,$$

The reception bandwidth can be increased by fabricating the reflectors with larger intervals (Figure 7b), or decreasing *γ* (Figure 7c) through increasing the frequency scanning bandwidth (*B_Ω_*), decreasing the scanning points (*N*), or reducing the sampling time (*Δt*). These four parameters also affect other aspects of system performance, such as the range sampling resolution (*Sr*), maximum measurement range (Lr=cN2BΩn), and signal-to-noise ratio (inversely proportional to *Δt*). The tradeoffs among the different aspects of system performances can be tuned to satisfy the requirements of different applications.

### 4.2. Number of Sensors

The maximum number of sensing units *N_C_* in the system is the ratio of maximum measurement range and the spatial resolution ($N_{c}=L_{r}/S_{R}$). Use $N_{c}$ to substitute its expression in Equation (11), and we obtain:

(12) $$N_{c}={\left(2B_{r}\Delta t\right)}^{-1},$$

Equation (12) indicates that Δ*t*, at the given, the larger the reception bandwidth, the smaller the number of sensors can be integrated into the system, and vice versa. For example, in the demonstration experiment in Section 3.4, the *N_c_* is about 2144, and the reception bandwidth is 6.5 Hz. When increasing the spacing between the sensing unit to 1 m, the reception bandwidth expands to 130 Hz. Accordingly, the *N_c_* reduces to 107 units. The loss from the sensing cable can also limit the *N_c_*, but this factor is not dominating when over several hertz reception bandwidth is required.

### 4.3. Delay in Signal Reading

There is a time delay between the waveform reading and the occurrence due to the frequency sweeping and signal post-processing. The delay is usually from milliseconds to seconds, which can be minimized by choosing a small number of modulation frequencies.

## 5. Conclusions

This paper presents a microwave photonics method for distributed acoustic sensing. This method encodes acoustic wave-induced strains along the sensing fiber in the microwave spectrum and demodulates them through a time–frequency joint method. The maximum measurable acoustic frequency is determined by the microwave frequency scanning rate and is independent of the spatial resolution and measurement range. The system successfully measured a sinusoidal signal of 16.7 kHz in frequency. The concept was further demonstrated using an optical fiber reflector array and a reference MI. Two PCCs were attached to the reflector array at different locations that were 0.5 m apart and driven by two sinusoidal voltages with different frequencies of 1700 Hz and 3430 Hz, respectively. The temporal strain signals read from the sensing fiber were reconstructed. The phase, frequency, and magnitude of the reconstructed temporal signal agree with those of the respective driving signal. The results show that the CMPI system can resolve 20 nε at kilohertz range when the gauge length is 10 cm and spatial resolution is 5 cm, indicating an excellent potential for DAS.

CMPI-based DAS technology enables flexibly choosing the set of sensing range, range resolution, and reception bandwidth based on the application requirements. Therefore, it has broad application prospects. For instance, the long-range and high-sensitivity capabilities allow its use in seismic sensing and downhole fracture detection. The centimeter-scale resolution capability makes it suitable for applications in the aerospace industry and medical care.

## Data Availability

Not applicable.

## References

[1] Walter F., Gräff D., Lindner F., Paitz P., Köpfli M., Chmiel M., Fichtner A. (2020). Distributed acoustic sensing of microseismic sources and wave propagation in glaciated terrain. Nat. Commun..

[2] Dou S., Lindsey N., Wagner A.M., Daley T.M., Freifeld B., Robertson M., Peterson J., Ulrich C., Martin E.R., Ajo-Franklin J.B. (2017). Distributed acoustic sensing for seismic monitoring of the near surface: A traffic-noise interferometry case study. Sci. Rep..

[3] Ferguson R.J., McDonald M.A., Basto D.J. (2020). Take the Eh? train: Distributed acoustic sensing (DAS) of commuter trains in a Canadian City. J. Appl. Geophys..

[4] Stajanca P., Chruscicki S., Homann T., Seifert S., Schmidt D., Habib A. (2018). Detection of leak-induced pipeline vibrations using fiber—Optic distributed acoustic sensing. Sensors.

[5] Barrias A., Casas J.R., Villalba S. (2016). A review of distributed optical fiber sensors for civil engineering applications. Sensors.

[6] Hartog A.H. (2017). An Introduction to Distributed Optical Fibre Sensors.

[7] Fernández-Ruiz M.R., Costa L., Martins H.F. (2019). Distributed acoustic sensing using chirped-pulse phase-sensitive OTDR technology. Sensors.

[8] Muanenda Y., Faralli S., Oton C.J., Di Pasquale F. (2018). Dynamic phase extraction in a modulated double-pulse ϕ-OTDR sensor using a stable homodyne demodulation in direct detection. Opt. Express.

[9] Zhu T., He Q., Xiao X., Bao X. (2013). Modulated pulses based distributed vibration sensing with high frequency response and spatial resolution. Opt. Express.

[10] Muanenda Y. (2018). Recent advances in distributed acoustic sensing based on phase-sensitive optical time domain reflectometry. J. Sensors.

[11] Wang Z., Zhang B., Xiong J., Fu Y., Lin S., Jiang J., Chen Y., Wu Y., Meng Q., Rao Y. (2019). Distributed acoustic sensing based on pulse-coding phase-sensitive OTDR. IEEE Internet Things J..

[12] Wang Z., Zhang L., Wang S., Xue N., Peng F., Fan M., Sun W., Qian X., Rao J., Rao Y. (2016). Coherent Φ-OTDR based on I/Q demodulation and homodyne detection. Opt. Express.

[13] Pastor-Graells J., Cortés L.R., Fernández-Ruiz M.R., Martins H., Azaña J., Martin-Lopez S., Gonzalez-Herraez M. (2017). SNR enhancement in high-resolution phase-sensitive OTDR systems using chirped pulse amplification concepts. Opt. Lett..

[14] Shan Y., Ji W., Wang Q., Cao L., Wang F., Zhang Y., Zhang X. (2018). Performance optimization for phase-sensitive OTDR sensing system based on multi-spatial resolution analysis. Sensors.

[15] Zinsou R., Liu X., Wang Y., Zhang J., Wang Y., Jin B. (2019). Recent progress in the performance enhancement of phase-sensitive OTDR vibration sensing systems. Sensors.

[16] Ding Z., Wang C., Liu K., Jiang J., Yang D., Pan G., Pu Z., Liu T. (2018). Distributed optical fiber sensors based on optical frequency domain reflectometry: A review. Sensors.

[17] Froggatt M., Moore J. (1998). High-spatial-resolution distributed strain measurement in optical fiber with Rayleigh scatter. Appl. Opt..

[18] Leviatan E., Eyal A. (2015). High resolution DAS via sinusoidal frequency scan OFDR (SFS-OFDR). Opt. Express.

[19] Shiloh L., Eyal A. (2017). Sinusoidal frequency scan OFDR with fast processing algorithm for distributed acoustic sensing. Opt. Express.

[20] Li H., Liu Q., Chen D., Deng Y., He Z. (2020). High-spatial-resolution fiber-optic distributed acoustic sensor based on Φ-OFDR with enhanced crosstalk suppression. Opt. Lett..

[21] Arbel D., Eyal A. (2014). Dynamic optical frequency domain reflectometry. Opt. Express.

[22] Hervás J., Ricchiuti A.L., Li W., Zhu N.H., Fernández-pousa C.R. (2017). Microwave photonics for optical fiber sensors. IEEE J. Sel. Top. Quantum Electron..

[23] Huang J., Lan X., Wang H., Yuan L., Xiao H. (2014). Optical carrier-based microwave interferometers for sensing application. Fiber Opt. Sens. Appl. XI.

[24] Hua L., Song Y., Cheng B., Zhu W., Zhang Q., Xiao H. (2017). Coherence-length-gated distributed optical fiber sensing based on microwave-photonic interferometry. Opt. Express.

[25] Liehr S., Wendt M., Krebber K. (2010). Distributed strain measurement in perfluorinated polymer optical fibres using optical frequency domain reflectometry. Meas. Sci. Technol..

[26] Clement J., Maestre H., Torregrosa G., Fernández-Pousa C.R. (2019). Incoherent optical frequency-domain reflectometry based on homodyne electro-optic downconversion for fiber-optic sensor interrogation. Sensors.

[27] Huang J., Lan X., Luo M., Xiao H. (2014). Spatially continuous distributed fiber optic sensing using optical carrier based microwave interferometry. Opt. Express.

[28] Liehr S., Nöther N., Krebber K. (2009). Incoherent optical frequency domain reflectometry and distributed strain detection in polymer optical fibers. Meas. Sci. Technol..

[29] Ricchiuti A.L., Hervas J., Barrera D., Sales S., Capmany J. (2014). Microwave photonics filtering technique for interrogating a very-weak fiber bragg grating cascade sensor. IEEE Photonics J..

[30] Bellido J.C., Peralta J.H., Madrigal J.M., Vicente H.M., Penalva G.T., Fernandez-Pousa C.R., Maicas S.S., Hervas J., Maestre H., Torregrosa G. (2018). Fast incoherent OFDR interrogation of FBG arrays using sparse radio frequency responses. J. Light. Technol..

[31] Cheng R., Xia L., Yan J., Zhou J., Wen Y., Rohollahnejad J. (2015). Radio frequency FBG-based interferometer for remote adaptive strain monitoring. IEEE Photonics Technol. Lett..

[32] Hua L., Zhu X., DeWolf S., Lei J., Zhang Q., Murdoch L., Xiao H. (2021). Phase demodulation by frequency chirping in coherence microwave photonic interferometry. IEEE J. Sel. Top. Quantum Electron..

[33] Redding B., Murray M.J., Donko A., Beresna M., Masoudi A., Brambilla G. (2020). Low-noise distributed acoustic sensing using enhanced backscattering fiber with ultra-low-loss point reflectors. Opt. Express.

[34] Wu M., Li C., Fan X., Liao C., He Z. (2020). Large-scale multiplexed weak reflector array fabricated with a femtosecond laser for a fiber-optic quasi-distributed acoustic sensing system. Opt. Lett..

[35] Zhou D., Dong Y., Yao J. (2020). Truly distributed and ultra-fast microwave photonic fiber-optic sensor. J. Light. Technol..

[36] Zhu X., Hua L., Tang J., Murdoch L., Xiao H. (2021). Microwave photonic reflectometry for dark-zone free distributed optical fiber sensing. Opt. Lett..

[37] Požar T., Gregorčič P., Možina J. (2011). A precise and wide-dynamic-range displacement-measuring homodyne quadrature laser interferometer. Appl. Phys. B.

[38] Cheng B., Song Y., Hua L., Xiao H. (2020). Fabrication and characterization of femtosecond laser induced microwave frequency photonic fiber grating. J. Light. Technol..

